# Effect of endometrial preparation protocols on the risk of ectopic pregnancy for frozen embryo transfer

**DOI:** 10.1038/s41598-021-97044-6

**Published:** 2021-08-31

**Authors:** Seung Chik Jwa, Masashi Takamura, Akira Kuwahara, Takeshi Kajihara, Osamu Ishihara

**Affiliations:** 1 Department of Obstetrics and Gynecology, Saitama Medical University, 38 Morohongo, Moroyama, Saitama 350-0495 Japan; 2 Department of Obstetrics and Gynecology, Graduate School of Biomedical Sciences, Tokushima University, Tokushima, Japan

**Keywords:** Diseases, Endocrinology, Medical research

## Abstract

Studies have consistently reported a significantly reduced incidence of ectopic pregnancy (EP) for frozen-thawed embryo transfer (ET) cycles compared with fresh cycles. However, only a few studies reported an association between endometrial preparation protocols on EP and results were conflicting. A registry-based retrospective cohort study of 153,354 clinical pregnancies following frozen single ETs between 2014 and 2017 were conducted, of which 792 cases of EP (0.52%) were reported. Blastocyst embryo transfers accounted for 87% of the total sample and were significantly associated with a decreased risk for EP compared with early cleavage ET (0.90% vs. 0.46%, adjusted OR = 0.50, 95% CI, 0.41 to 0.60). Compared with natural cycles, hormone replacement cycles (HRC) demonstrated a similar risk for EP (0.53% vs. 0.47%, adjusted OR = 1.12, 95% CI, 0.89 to 1.42). Subgroup analysis with or without tubal factor infertility and early cleavage/blastocyst ETs demonstrated similar non-significant associations. Endometrial preparation protocols using clomiphene (CC) were associated with a significantly increased risk for EP (1.12%, adjusted OR = 2.34; 95% CI, 1.38 to 3.98). These findings suggest that HRC and natural cycles had a similar risk for EP. Endometrial preparation using CC was associated with an increased risk of EP in frozen embryo transfer cycles.

## Introduction

Despite significant improvements in in vitro fertilization (IVF) techniques, ectopic pregnancy (EP) remains a serious problem in assisted reproductive technology (ART), occurring 2–5%^1–3^ higher than in the general population (1–2%)^4–6^. Various factors in ART are associated with EP, such as the number of embryos transferred^7^, assisted hatching^8^ and embryo stage at embryo transfer (ET)^9^. Further, patient related factors such as tubal factor infertility diagnosis, endometriosis, and smoking are also associated with the risk of EP^10–13^. Studies have consistently reported a significantly reduced incidence of EP for frozen-thawed ET cycles compared with fresh cycles^7,14–16^. The lower risk of EP for frozen-thawed ET cycles may reflect the more physiological hormonal environment compared with stimulated cycles^1,17^.

Several endometrial preparation protocols, such as natural ovulatory cycle, hormone replacement cycles (HRC) and ovarian stimulation using exogeneous gonadotropin (Gn), clomiphene (CC) and letrozole, have been applied in frozen-thawed ET cycles. Although these cycles were more equivalent to the physiological hormonal environment than fresh cycles, because HRC and ovarian stimulation cycles use medication, the hormonal environments are different among different endometrial preparation protocols.

To date, only a few studies reported an association between endometrial preparation protocols on EP and results were conflicting^12,18,19^. In China, recent studies investigating the effect of endometrial thickness on EP in frozen-thawed ET cycles reported that the EP rate for HRC cycles was more than double the rate for natural cycles (4.7% vs. 2.1%, respectively)^12^. However, double embryo transfer and early cleavage stage ET in that study accounted for 86% and 84.9% of the cases examined, respectively, both of which could affect the incidence of EP. The differences in ART practice and extremely high EP rate (3.15%) limit the generalization of their study results^20^.

In Japan, single embryo transfer (SET) has accounted for more than 80% of ART cases because of the recommendation by the Japan Society of Obstetrics and Gynecology (JSOG) in 2008^21^. Furthermore, the mandatory reporting system of the Japanese ART registry is a cycle-based format, with almost all treatment cycles registered. Since 2014, endometrial preparation protocols using HRC were collected in this registry. The current study aimed to evaluate the risk of EPs for frozen-thawed single ET cycles on the basis of different endometrial preparation protocols.

## Results

Baseline characteristics of the sample population stratified by EP are shown in Table 1. Among 153,354 clinical pregnancies and 792 cases of EP (0.52%) were reported. Mean maternal age was 36 years, which was similar in the EP and non-EP groups. EP was significantly more frequent in patients exhibiting tubal factor infertility (0.95%) compared with cases without tubal factor infertility (0.44%) (*P* < 0.001). Similarly, endometriosis was significantly associated with an increased incidence of EP, while infertile couples with male factor and unexplained infertility had a significantly lower incidence of EP compared with infertile couples without these diagnoses. Among endometrial preparation protocols, the EP rate was highest in cycles using CC (1.12%), followed by HRC (0.53%), and the lowest rate was in the natural cycle (0.47%) group; with overall analysis showing a significant association between endometrial preparation protocols and EP (*P* = 0.004). The EP rate was significantly higher for early cleavage stage ET (0.90%) than blastocyst ET (0.46%) (*P* < 0.001).

**Table 1:** Baseline characteristics of the sample population stratified by ectopic pregnancies. (n = 153,354).

Characteristics	Non-ectopic pregnancy	Ectopic pregnancy	*P* value^a^
**Maternal age, (year)**	36.0 (4.1)	36.1 (4.0)	0.28
< 30	10,156 (99.6)	46 (0.45)	0.71
30–34	44,455 (99.5)	229 (0.51)	
35–39	65,840 (99.5)	354 (0.53)	
≥ 40	32,111 (99.5)	163 (0.51)	
**Tubal factor infertility**			
Yes	22,894 (99.1)	220 (0.95)	< 0.001
No	129,668 (99.6)	572 (0.44)	
**Endometriosis**			
Yes	10,576 (99.3)	80 (0.75)	< 0.001
No	141,986 (99.5)	712 (0.50)	
**Antisperm antibody**			
Yes	772 (99.1)	7 (0.90)	0.14
No	151,790 (99.5)	785 (0.51)	
**Male factor**			
Yes	42,900 (99.6)	177 (0.41)	< 0.001
No	109,662 (99.4)	615 (0.56)	
**PCOS/anovulation**			
Yes	6515 (99.6)	29 (0.44)	0.40
No	146,047 (99.5)	763 (0.52)	
**Others**			
Yes	20,772 (99.4)	127 (0.61)	0.048
No	131,790 (99.5)	665 (0.50)	
**Unexplained**			
Yes	67,027 (99.6)	283 (0.42)	< 0.001
No	85,535 (99.4)	509 (0.59)	
**Endometrial preparation protocol**			
Natural	45,281 (99.5)	216 (0.47)	0.004
HRC	100,275 (99.5)	536 (0.53)	
Clomiphene	1586 (98.9)	18 (1.12)	
Letrozole	4246 (99.6)	16 (0.38)	
Gonadotropin	1174 (99.5)	6 (0.51)	
**Embryo stage at transfer**			
Early cleavage	19,697 (99.1)	178 (0.90)	< 0.001
Blastocyst	132,865 (99.5)	614 (0.46)	
**Assisted hatching**^**b**^			
( +)	104,201 (99.5)	525 (0.50)	0.22
( −)	48,358 (99.5)	267 (0.55)	
**Luteal support**^**c**^			
None	8576 (99.6)	38 (0.44)	0.32
Progesterone	36,916 (99.6)	156 (0.42)	0.003
hCG	6184 (99.4)	38 (0.61)	0.29
Progesterone + hCG	7297 (99.4)	48 (0.65)	0.09
Estrogen + Progesterone	93,761 (99.5)	518 (0.55)	0.02
Others	3709 (99.5)	20 (0.54)	0.86
**Year**			
2014	29,725 (99.5)	163 (0.55)	0.64
2015	35,716 (99.5)	194 (0.54)	
2016	41,340 (99.5)	202 (0.49)	
2017	45,781 (99.5)	233 (0.51)	

Data are presented as mean (SD) for continuous variables and n (%) for dichotomous variables. Percentages are presented in the rows for the purpose of comparison.

*hCG* human chorionic gonadotropin, *HRC* hormone replacement cycle, *PCOS* polycystic ovarian syndrome.

^a^*P* values were assessed with the use of the χ^2^ or Student's t test.

^b^3 cases were missing in the variable.

^c^Multiple answers were allowed.

Associations between baseline characteristics and endometrial preparation protocols are shown in Supplemental Table 1. Maternal age was highest in the natural cycle group (mean = 36.8, standard deviation [SD] = 3.9), while the HRC (mean = 35.4, SD = 4.1) and CC (mean = 35.4, SD = 4.1) groups had the youngest ages. The HRC and letrozole groups had the highest prevalence of polycystic ovarian syndrome (PCOS) (5.5% and 8.7%, respectively). Blastocyst stage ET was performed in more than 85% of patients in the natural, HRC, letrozole and Gn groups. Early cleavage stage ET was most frequent in the CC group (26.6%).

Crude and adjusted ORs for EP after various ART treatments are shown in Table 2. The crude and adjusted ORs for HRC (adjusted OR = 1.12, 95% CI, 0.89 to 1.42) were not significantly different compared with the natural cycle. However, ovarian stimulation using CC demonstrated a significant higher adjusted OR (adjusted OR = 2.34, 95% CI, 1.38 to 3.98) for EP compared with the natural cycle. Blastocyst transfer was significantly associated with a decreased risk for EP (adjusted OR = 0.50, 95% CI, 0.41 to 0.60) compared with early cleavage ET.

**Table 2:** Risk of ectopic pregnancy for ART treatment using frozen single embryo transfer cycles (n = 153,354).

	Crude OR (95% CI)	Adjusted OR (95% CI)^a^
**Endometrial preparation protocol**		
Natural	Reference	Reference
HRC	1.12 (0.89 to 1.41)	1.12 (0.89 to 1.42)
Clomiphene	**2.38 (1.41 to 4.01)**	**2.34 (1.38 to 3.98)**
Letrozole	0.79 (0.49 to 1.29)	0.81 (0.49 to 1.32)
Gonadotropin	1.07 (0.43 to 2.67)	1.02 (0.41 to 2.54)
**Embryo stage at transfer**		
Early cleavage	Reference	Reference
Blastocyst	**0.51 (0.43 to 0.61)**	**0.50 (0.41 to 0.60)**
**Assisted hatching**		
( −)	Reference	Reference
( +)	1.10 (0.89 to 1.35)	1.08 (0.88 to 1.33)

Significantly increased or reduced odds are indicated by boldface.

*CI* confidence interval, *HRC* hormone replacement cycle, *OR* odds ratio.

^a^Adjusted for maternal age, infertility diagnosis of tubal factor, endometriosis, male factor, other or unexplained and year.

Results of subgroup analysis for patients with or without tubal factor infertility, excluding both tubal factor and endometriosis, and early cleavage and blastocyst ETs are shown in Table 3. For the subgroups of tubal factor infertility (n = 23,114) and early cleavage ETs (n = 19,731), there were no significant associations between endometrial preparation protocols and the risk of EP. Conversely, for subgroups without tubal factor infertility diagnosis (n = 130,340 cycles), excluding tubal factor and endometriosis (n = 121,341 cycles) and blastocyst ET cycles (n = 133,479 cycles), significant associations were found for endometrial preparations using CC and the risk of EP; without tubal infertility (OR = 2.31, 95% CI, 1.33 to 4.01), excluding tubal factor and endometriosis (OR = 2.14, 95% CI, 1.18 to 3.86) and with blastocyst ET (OR = 2.66, 95% CI, 1.50 to 4.72), compared with natural cycles.

**Table 3:** Subgroup analysis of crude and adjusted ORs for ectopic pregnancy with endometrial preparation protocols.

	Crude OR (95% Cl)	Adjusted OR (95% Cl)
**Tubal factor infertility (n = 23,114)**^**a**^		
Natural	Reference	Reference
HRC	1.32 (0.93 to 1.87)	1.37 (0.95 to 1.98)
Clomiphene	2.35 (0.81 to 6.72)	2.29 (0.80 to 6.55)
Letrozole	0.86 (0.38 to 1.93)	0.86 (0.38 to 1.93)
Gonadotropin	0.57 (0.09 to 3.73)	0.59 (0.09 to 3.82)
**Without tubal factor infertility (n = 130,240)**^**a**^		
Natural	Reference	Reference
HRC	1.05 (0.83 to 1.34)	1.03 (0.82 to 1.30)
Clomiphene	**2.42 (1.42 to 4.14)**	**2.31 (1.33 to 4.01)**
Letrozole	0.78 (0.48 to 1.26)	0.79 (0.49 to 1.29)
Gonadotropin	1.25 (0.47 to 3.31)	1.22 (0.46 to 3.23)
**Without tubal factor and endometriosis (n = 121,341)**^**b**^		
Natural	Reference	Reference
HRC	1.002 (0.79 to 1.27)	1.006 (0.79 to 1.28)
Clomiphene	**2.22 (1.26 to 3.93)**	**2.14 (1.18 to 3.86)**
Letrozole	0.73 (0.46 to 1.17)	0.73 (0.46 to 1.18)
Gonadotropin	0.80 (0.24 to 2.65)	0.79 (0.24 to 2.65)
**Early cleavage (n = 19,731)**^**c**^		
Natural	Reference	Reference
HRC	0.75 (0.49 to 1.15)	0.74 (0.49 to 1.11)
Clomiphene	1.07 (0.41 to 2.78)	1.13 (0.42 to 3.03)
Letrozole	0.98 (0.45 to 2.17)	0.99 (0.45 to 2.17)
Gonadotropin	NA	NA
**Blastocyst (n = 133,479)**^**c**^		
Natural	Reference	Reference
HRC	1.20 (0.98 to 1.46)	1.20 (0.98 to 1.47)
Clomiphene	**2.75 (1.58 to 4.80)**	**2.66 (1.50 to 4.72)**
Letrozole	0.76 (0.43 to 1.36)	0.78 (0.43 to 1.40)
Gonadotropin	1.44 (0.56 to 3.71)	1.38 (0.53 to 3.54)

Significantly increased or reduced odds are indicated by boldface.

*CI* confidence interval, *HRC* hormone replacement cycle, *NA* not available, *OR* odds ratio.

^a^Adjusted for maternal age, infertility diagnosis of endometriosis, male factor, other or unexplained and year.

^b^Adjusted for maternal age, infertility diagnosis of male factor, other or unexplained and year.

^c^Adjusted for maternal age, infertility diagnosis of tubal factor, endometriosis, male factor, other or unexplained and year.

## Discussion

This study analyzed a large nationally-representative ART register for frozen-thawed single ETs in Japan, and found an EP rate of 0.52%, which was much smaller than the recently reported EP rate for fresh single ET cycles (1.46%) using the same Japanese registry^22^. Single ET using blastocysts resulted in an almost 50% reduction in the risk of EP compared with early cleavage stage ET. There was no significant difference between natural cycles and HRC for the risk of EP. The use of CC during endometrial preparation was associated with a significantly increased risk for EP. To date, this is the largest study reporting the effect of endometrial preparation protocols on the risk of EP in frozen single ET cycles.

The lower risk for EP in frozen-thawed blastocyst ET cycles compared with fresh ET cycles was first reported by Ishihara et al. using the Japanese ART registry^16^. Since then, several studies have reported a similar risk reduction for EP in frozen compared with fresh ET cycles^14,23^. Recent meta-analysis investigated the risks of EP in frozen-thawed versus fresh blastocyst ETs using fourteen retrospective studies (n = 251,762 cycles) and demonstrated that the EP rate for fresh single blastocyst ET cycles (1.2%) was significantly higher than that for frozen-thawed blastocyst ET cycles (0.80%)^23^. One of the differences between fresh and frozen cycles is the ovarian stimulation. Our previous study of the effect of ovarian stimulation protocols on the risk of EP demonstrated that the EP rate for natural (i.e., unstimulated) IVF cycles was 0.47%, significantly lower than the rate for stimulated cycles (1.47–2.18%). Interestingly, the EP rate for unstimulated fresh cycles (0.47%) was similar to the EP rate for all frozen-thawed cycles (0.52%) examined in the current study. These results indicate that ovarian stimulation may mediate the elevated risk of EP for the fresh cycles.

For the frozen-thawed cycles in our study, the risk of EP for blastocyst ET was almost halved compared with early cleavage stage ET, consistent with previous studies. Recent meta-analysis of 22 studies (143,643 pregnancies) investigated the effect of embryo stage at transfer on EP and found that the risk of EP was significantly lower for blastocyst ET than early cleavage stage ET for both fresh (relative risk [RR] = 0.78, 95% CI, 0.69 to 0.88) and frozen-thawed ETs (RR = 0.43, 95% CI, 0.36 to 0.51)^9^. It was suggested that the time period for implantation would be reduced for blastocysts compared with early cleavage stage embryos after ET, and would therefore provide a lower chance to migrate to fallopian tubes^24^. Furthermore, uterine contraction 3–5 days after ovulation would direct embryos from the uterine cervix towards the fundus, so early cleavage stage embryos would be more likely to migrate to the fallopian tubes^25^. Another potential explanation for the decreased risk of EP for blastocyst embryos maybe the larger size of blastocysts compared with cleavage stage embryos, preventing migration to fallopian tubes^26^.

Although many studies have investigated endometrial preparation protocols and pregnancy and live birth rates^27–29^, only a limited number of studies with conflicting results have investigated endometrial preparation protocols and the risk of EP^12,18,19^. Importantly, these studies did not specifically investigate endometrial preparation protocols and targeted other factors. One recent study investigated the effect of endometrial thickness on EP, using 17,244 pregnancies following frozen ETs from a single institution in China^12^. The study demonstrated that HRC was significantly associated with EP compared with the modified natural cycle (202/4294 [4.7%] in HRC cycles vs. 105/4941 [2.1%] in modified natural cycles, adjusted OR = 2.25, 95% CI, 1.76 to 2.87). However, double embryo transfer was performed in 86% of the total cycles, and early cleavage ETs predominated (84.9%). Because of these characteristics, the EP rate was very high (3.15%) in this Chinese sample, so caution is required with the reported association of HRC with EP. However, another Chinese study investigating the association between body mass index and EP using retrospective data (n = 16,378 pregnancies) demonstrated no significant associations between endometrial preparation protocols and the risk of EP for frozen cycles^18^. Furthermore, a retrospective analysis of clinical pregnancies in Italy following single blastocyst ETs with different endometrial preparation protocols demonstrated a significantly higher EP rate for natural cycles (6/561, 3.28%) than HRC (4/585, 1.83%) and modified natural cycles (3/1749, 0.4%)^19^. Our study, using the largest sample of subjects with single frozen-thawed ET cycles demonstrated that the risk of EP did not differ for HRC compared with natural cycles.

Nevertheless, patient-related factors such as infertility diagnosis would still impact on the incidence of EP in frozen-thawed ET cycles. Boynukalin et al. investigated the risk of EP in 13,261 fresh and frozen-thawed ET cycles without tubal infertility cases and found that the risk of EP was the lowest in frozen-thawed ET cycles with embryos from previous freeze-all cycles (eFET, 0.9%) than fresh ET cycles (1.6%) and frozen-thawed ET with surplus embryos from a previous fresh cycle (1%)^30^. Interestingly, day 3 ET in eFET group was a significantly lower risk for EP than day 5 ET (1/360 [0.03%] vs. 15/1357 [1.1%], *P* < 0.05). Thus, we have conducted an additional analysis using the subgroup excluding tubal factor infertility diagnosis and endometriosis and blastocyst ET demonstrated still a significantly decreased risk for EP (adjusted OR = 0.60, 95% CI, 0.49 to 0.75). Thus, patient-related factors and previous freeze-all in fresh cycles might impact on the risk of EP in frozen-thawed ET cycles.

The use of CC was associated with an increased risk of EP. Interestingly, our previous study investigating the effect of ovarian stimulation on the risk of EP for fresh cycles also demonstrated a significantly elevated risk of EP for ovarian stimulation using CC; EP rate of cycles using CC was the highest (1.81–2.18%) compared with other ovarian stimulation regimens (0.47–1.57%)^22^. One potential reason for the elevated risk of EP from cycles using CC was endometrial thickness. It is well known that ovulation induction using CC often results in a thinner endometrium, and it was reported that endometrial thickness in the mid-luteal phase was significantly thinner in women receiving CC compared with control cycles without CC^31^. Thinner endometrium during frozen-thawed ET cycles was reported to be associated with an increased risk for EP^12^. Furthermore, the effect of CC on endometrial receptivity^32^ and apoptotic changes in fallopian tube epithelial cells have been reported^33^. Such changes in cycles using CC may result in reduced probability of embryo implantation in the uterine endometrium compared with the probability of EP per clinical pregnancy. Clinical pregnancy rates for frozen single ETs in the Japanese registry were much lower for CC cycles (27.0%) than natural (37.0%), HRC (34.3%), letrozole (42.3%) and Gn (32.9%) protocols (data not shown).

One strength of the current study was the large number of frozen-thawed single ET cycles, enabling the investigation of relatively rare but serious events of EP on the basis of various endometrial protocols. Because the Japanese ART registry system involves a mandatory registration system in conjunction with governmental subsidies, all treatment cycles are registered in the system, so sampling bias would be less likely. Furthermore, with the high single ET rate (more than 80% of ETs), the current study provides an EP rate using autologous embryos and eliminates the effect of number of embryos transferred. However, several limitations also exist in the study. The study was retrospective, so the medical indication for selecting endometrial preparation protocols was unknown. Thus, although we have adjusted for factors such as age and several infertility diagnosis, which were associated with endometrial preparation protocols (Supplemental Table 1), unmeasured confounders such as smoking, body mass index and ovarian reserve may impact the results. Furthermore, the current study lacked information on endometrial thickness, and estrogen and progesterone levels during the cycles, so we were unable to elucidate the possible mediating factors between endometrial preparation protocols and the risk of EP, especially in cycles using CC. Lastly, because 97.8% of the population in Japan is currently Japanese^34^, we cannot consider the effects of race and ethnicity. Similarly, advanced age is the major contributor for the increasing number of ART patients in Japan, and the mean maternal age of the current study was relatively high (36 years). Thus, to confirm the present findings, it is essential that prospective studies include potential confounders and mediators in different populations.

In conclusion, using the nationally representative ART data for a large Japanese sample with a high single ET rate, our study demonstrated that endometrial preparation protocols using HRC had a similar risk for EP compared with natural cycles. Given the increasing number of frozen-thawed ETs worldwide, the current results are reassuring for the use of HRC in terms of the risks of EP. Further research would be necessary to elucidate potential mediating factors, especially for the use of CC for endometrial preparation and the risk of EP.

## Materials and methods

### Sample selection

This was a registry-based retrospective cohort study using the Japanese national ART registry managed by the JSOG. The ART facilities in Japan have to register their treatment cycles for patients to receive government subsidies. The registry consists of cycle-specific information, including patient background and treatment information. Because treatment using donated oocytes is not allowed in Japan, all ET cycles are autologous. Furthermore, preimplantation genetic testing for chromosomal aneuploidy was not practiced in Japan during the study period. This study was approved by the institutional review board of Saitama Medical University (Approval number 916, April. 2020) and the ethics committee of the JSOG (Approval number 2020-6, September. 2020). The study was conducted in accordance with Japanese law and the STROBE Guidelines. Informed consent was waived by the institutional review board of Saitama Medical University since this study used anonymized data.

**Figure 1:**
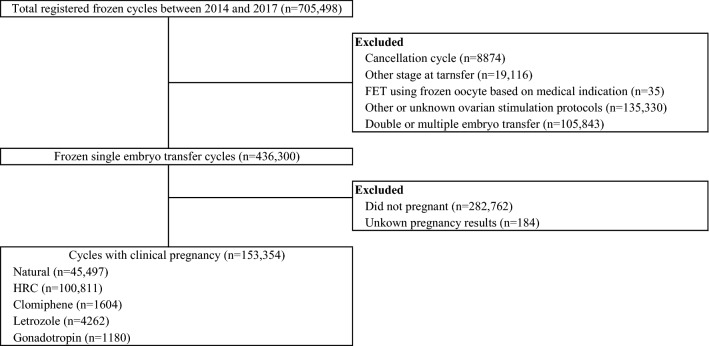
Flow diagram of cohort selection.

A detailed flow diagram of sample selection for analysis is shown in Fig. 1. We included clinical pregnancies after frozen-thawed single ET cycles between 2014 and 2017. Among 705,498 registered cycles, we excluded cancellation cycles (n = 8874), ET cycles with embryos transferred on days other than early cleavage and blastocyst stage (n = 19,116), cycles using frozen oocytes based on medical indication, such as fertility preservation for cancer patients (n = 35), other or unknown endometrial preparation protocols (n = 135,330), and double or multiple frozen-thawed ET cycles (n = 105,843). In the remaining 436,300 single ET cycles, 153,354 clinical pregnancies with known pregnancy outcomes were reported (clinical pregnancy rate = 35.1%), and EP was reported in 792 of these cases (0.52%).

Endometrial preparation protocols consisted of natural cycle (i.e., unstimulated), HRC, ovarian stimulation using CC, letrozole and exogeneous Gn. Both endometrial preparation protocols using CC and letrozole included cycles with exogeneous Gn. We also used infertility diagnosis, embryo stage at transfer (early cleavage or blastocyst), assisted hatching and luteal phase support from the registry.

The definition of clinical pregnancy in the registry was the confirmation of a uterine gestational sac. The definition of EP was the confirmation of a gestational sac outside the uterine cavity (visualized by ultrasound) and also included clinically diagnosed cases in each ART facility, such as the combination of elevated serum human chorionic gonadotropin (hCG) levels without detection of a gestational sac. Heterotopic pregnancy, defined as an intrauterine pregnancy co-existing with an extrauterine gestational sac, was reported in 3 cases. In this study, heterotopic cases were defined as positive for the outcome, although the etiology for the occurrence of heterotopic pregnancy following single ET was unknown.

### Statistical analysis

We first compared baseline characteristics and treatment information according to EP and non-EP groups using the chi-squared or Student’s t-test. Odds ratios (ORs) and 95% confidence intervals (95% CIs) of ART treatment, including endometrial preparation protocols, embryo stage at transfer and assisted hatching for EP, were calculated using generalized estimating equations with robust variance estimation adjusting for correlations within ART facilities. Confounders adjusted for the analysis were maternal age (categorized into 5-year age groups), tubal factor infertility diagnosis, male factor infertility, endometriosis and the year of registered cycles, following previously reported associations^12^. Furthermore, we adjusted for the diagnosis of other or unexplained causes of infertility because of the significant associations between these variables and incidence of EP (Table 1). Furthermore, we conducted several subgroup analyses as follows using generalized estimating equations with robust variance estimation adjusting for correlations within ART facilities; (1) with or without tubal factor infertility, (2) excluding both tubal factor infertility and endometriosis, (3) early cleavage and blastocyst ETs. The statistical adjustment was conducted for the same variables described above other than excluded/stratified variables for the subgroups analysis. All analyses were conducted using the STATA MP statistical package, version 16.1 (Stata, College Station, TX, USA). A two-tailed valued of *P* < 0.05 was considered statistically significant.

## Supplementary Information

Supplementary Table 1.

## Data Availability

The datasets analyzed during the current study are not publicly available since the datasets include special care-required personal information but are available from the corresponding author on reasonable request.
