# Supplementary material for: Effect of endometrial preparation protocols on the risk of ectopic pregnancy for frozen embryo transfer
Source: Sci Rep. 2021 Aug 31;11:17453. doi: 10.1038/s41598-021-97044-6 (PMC8408134; doi:10.1038/s41598-021-97044-6)
Supplement: Supplementary file 1 — Supplementary Table 1. [file 41598_2021_97044_MOESM1_ESM.docx]

Supplementary material

**Effect of endometrial preparation protocols on the risk of ectopic pregnancy for frozen embryo transfer**

Seung Chik Jwa, Masashi Takamura, Akira Kuwahara, Takeshi Kajihara, and Osamu Ishihara

| **Supplemental Table 1. Baseline characteristics of the sample population stratified by endometrial preparation protocols (n=153,354)^a^.** | | | | | |  |
| --- | --- | --- | --- | --- | --- | --- |
| Characteristics | Natural (n=45,497) | HRC (n=100,811) | Clomiphene (n=1,604) | Letrozole (n=4,262) | Gonadotropin (n=1,180) | P value^b^ |
| Maternal age, (year) | 36.8 (3.9) | 35.5 (4.1) | 35.4 (4.1) | 36.6 (3.9) | 35.6 (4.0) | <0.001 |
| <30 | 1812 (4.0) | 8034 (8.0) | 136 (8.5) | 146 (3.4) | 74 (6.3) | <0.001 |
| 30-34 | 10694 (23.5) | 31978 (31.7) | 518 (32.3) | 1129 (26.5) | 365 (30.9) |  |
| 35-39 | 20606 (45.3) | 42446 (42.1) | 676 (42.1) | 1928 (45.2) | 538 (45.6) |  |
| ≥40 | 12385 (27.2) | 18353 (18.2) | 274 (17.1) | 1059 (24.9) | 203 (17.2) |  |
| Infertility diagnosis^c^ |  |  |  |  |  |  |
| Tubal factor | 6893 (15.2) | 15184 (15.1) | 220 (13.7) | 593 (13.9) | 224 (19.0) | <0.001 |
| Endometriosis | 2713 (6.0) | 7641 (7.6) | 105 (6.6) | 115 (2.7) | 82 (7.0) | <0.001 |
| Antisperm antibody | 176 (0.39) | 581 (0.58) | 11 (0.69) | 5 (0.12) | 6 (0.51) | <0.001 |
| Male factor | 10955 (24.1) | 30567 (30.3) | 442 (27.6) | 771 (18.1) | 342 (29.0) | <0.001 |
| PCOS/anovulation | 630 (1.4) | 5503 (5.5) | 36 (2.24) | 357 (8.4) | 18 (1.53) | <0.001 |
| Others | 4497 (9.9) | 15575 (15.5) | 376 (23.4) | 258 (6.1) | 193 (16.4) | <0.001 |
| Unexplained | 23724 (52.1) | 40114 (39.8) | 572 (35.7) | 2458 (57.7) | 442 (37.5) | <0.001 |
| Embryo stage at transfer |  |  |  |  |  |  |
| Early cleavage | 4652 (10.2) | 14281 (14.2) | 427 (26.6) | 371 (8.7) | 144 (12.2) | <0.001 |
| Blastocyst | 40845 (89.8) | 86530 (85.8) | 1177 (73.4) | 3891 (91.3) | 1036 (87.8) |  |
| Assisted hatching^d^ |  |  |  |  |  |  |
| (+) | 30367 (66.8) | 69900 (69.3) | 650 (40.5) | 2993 (70.2) | 816 (69.2) | <0.001 |
| (－) | 15127 (33.3) | 30911 (30.7) | 954 (59.5) | 1269 (29.8) | 364 (30.9) |  |
| Luteal support^c^ |  |  |  |  |  |  |
| None | 7104 (15.6) | 1088 (1.1) | 215 (13.4) | 189 (4.4) | 18 (1.5) | <0.001 |
| Progesterone | 36,916 (52.8) | 9440 (9.4) | 347 (21.6) | 3131 (73.5) | 126 (10.7) | <0.001 |
| hCG | 3458 (7.6) | 2049 (2.0) | 164(10.2) | 35 (0.82) | 516 (43.7) | <0.001 |
| Progesterone +hCG | 5126 (11.3) | 1278 (1.27) | 594 (37.0) | 67 (1.6) | 280 (23.7) | <0.001 |
| Estrogen+Progesterone | 6083 (13.4) | 86510 (85.8) | 310 (19.3) | 845 (19.8) | 531 (45.0) | <0.001 |
| Others | 764 (1.7) | 2912 (2.9) | 12 (0.75) | 19 (0.45) | 22 (1.86) | <0.001 |
| Year |  |  |  |  |  |  |
| 2014 | 9494 (20.9) | 18918 (18.8) | 499 (31.1) | 794 (18.6) | 183 (15.5) | <0.001 |
| 2015 | 10677 (23.5) | 23624 (23.4) | 372 (23.2) | 940 (22.1) | 297 (25.2) |  |
| 2016 | 12342 (27.1) | 27352 (27.1) | 385 (24.0) | 1122 (26.3) | 341 (28.9) |  |
| 2017 | 12984 (28.5) | 30917 (30.7) | 348 (21.7) | 1406 (33.0) | 259 (30.4) |  |
| ^a^Data are presented as mean (SD) for continuous variables and n (%) for dichotomous variables. Percentages are presented in rows for the purpose of comparison. | | | | | | |
| ^b^P values were assessed with the use of the χ^2^ or Student's t test. | | | | | | |
| ^c^Multiple answers were allowed. |  |  |  |  |  |  |
| ^d^3 cases were missing in the variable. | |  |  |  |  |  |
| HRC, hormone replacement cycle; PCOS, polycystic ovarian syndrome. | | | | | | |
